# Pathological characterization of T2*-weighted MRI contrast in the striatum of Huntington’s disease patients

**DOI:** 10.1016/j.nicl.2020.102498

**Published:** 2020-11-10

**Authors:** Marjolein Bulk, Ingrid Hegeman-Kleinn, Boyd Kenkhuis, Ernst Suidgeest, Willeke van Roon-Mom, Jan Lewerenz, Sjoerd van Duinen, Itamar Ronen, Louise van der Weerd

**Affiliations:** a Department of Radiology, Leiden University Medical Center, Leiden, the Netherlands; b Department of Human Genetics, Leiden University Medical Center, Leiden, the Netherlands; c Department of Pathology, Leiden University Medical Center, Leiden, the Netherlands; d Department of Neurology, Ulm University Hospital, Ulm, Germany

**Keywords:** MRI, Iron, Huntington’s disease, Microglia, Astrocytes, Striatum

## Abstract

Previous MRI studies consistently reported iron accumulation within the striatum of patients with Huntington’s disease (HD). However, the pattern and origin of iron accumulation is poorly understood. This study aimed to characterize the histopathological correlates of iron-sensitive *ex vivo* MRI contrast change in HD brains. To this end, T2*-weighted 7T MRI was performed on postmortem tissue of the striatum of three control subjects and 10 HD patients followed by histological examination. In addition, formalin-fixed paraffin-embedded material of three control subjects and 14 HD patients was selected for only histology to identify the cellular localization of iron using stainings for iron, myelin, microglia and astrocytes. As expected HD striata showed prominent atrophy. Compared to controls, the striatum of HD patients was in general more hypointense on T2*-weighted high-field MRI and showed a more intense histopathological staining for iron. In addition, T2*-weighted MRI identified large focal hypointensities within the striatum of HD patients. Upon histological examination, these large focal hypointensities frequently colocalized with enlarged perivascular spaces and iron was found within the vessel wall and reactive astrocytes. In conclusion, we show that the striatum of HD patients has a distinctive phenotype on T2*-weighted MRI compared to control subjects. On *ex vivo* MRI, these contrast changes are heavily biased by enlarged perivascular spaces from which it is currently unknown whether this is a fixation artefact or a disease specific observation. Clinically, the observation of iron within reactive astrocytes is of importance for the interpretation and understanding of the potential underlying mechanisms of T2*-weighted MRI results in HD patients.

## Highlights

• HD striatum is characterized by hypointensities on ex vivo T2*-weighted MRI.
• Ex vivo MRI contrast changes colocalize with iron and enlarged perivascular spaces.
• In vivo validation of enlarged perivascular spaces in HD is needed.
• Iron is predominantly found within reactive astrocytes in the HD striatum.

## 1 Introduction

Huntington’s disease (HD) is an autosomal dominant neurodegenerative disorder caused by an expanded cytosine-adenine-guanine (CAG) trinucleotide repeat in the Huntingtin (*HTT*) gene (Macdonald, 1993). The disease-causing HTT alleles show > 39 CAG repeats in exon 1, with disease onset being earlier and progression faster the larger the number of CAG repeats is (Bates et al., 2015). Clinically, HD is characterized by a variety of motor disturbances (typically chorea and dystonia), cognitive decline and behavioural changes (Roos, 2010). The histopathological correlate of these signs and symptoms is progressive neuronal loss, especially of the medium spiny neurons of the striatum, followed by atrophy. During disease progression, other regions including the white matter and cortex are also affected, eventually resulting in global brain atrophy (Vonsattel et al., 2011).

Although HD has a clear monogenetic origin, the pathological cascade that leads to neuronal death is complicated, affecting many cell types and biological processes, and non-invasive methods to accurately monitor disease progressions *in vivo* remain to be developed. In addition, HD is one of the many neurodegenerative diseases in which increased levels of iron have been reported in the striatum, the brain region most severely affected (DEXTER et al., 1991, Domínguez D et al., 2016, Muller and Leavitt, 2014, Rosas et al., 2012). Iron is essential for many fundamental biological processes including oxygen transport, DNA synthesis, mitochondrial respiration in general, as well as myelin and neurotransmitter synthesis and metabolism in the brain specifically. However, due to its ability to catalyze the generation of reaction oxygen species via the Fenton reaction, iron levels have to be tightly controlled to prevent oxidative stress and ultimately neuronal loss (Muller and Leavitt, 2014, Ward et al., 2014, Zecca et al., 2004).

In HD the largest increase in iron is reported in the basal ganglia, and more specifically in the striatum (Domínguez D et al., 2016, Rosas et al., 2012, Bartzokis et al., 2007, Dumas et al., 2012, Jurgens et al., 2010, Sánchez-Castañeda et al., 2013, van Bergen et al., 2016, Vymazal et al., 2007). Due to the magnetic properties of iron, its presence affects the homogeneity of the local magnetic field in tissue, and hence contributes to MRI contrast, especially on T2*-weighted images (Chavhan et al., 2009). Previous neuroimaging studies have used a range of iron-sensitive MRI techniques to analyze cerebral iron accumulation in HD. These studies consistently reported MRI contrast changes, hypointensities and increased susceptibility values, indicative of iron accumulation in the basal ganglia (Domínguez D et al., 2016, Rosas et al., 2012, Bartzokis et al., 2007, Dumas et al., 2012, Jurgens et al., 2010, Sánchez-Castañeda et al., 2013, van Bergen et al., 2016, Vymazal et al., 2007). This effect has already been found in premanifest HD gene-carriers (Domínguez D et al., 2016, van Bergen et al., 2016), showing positive correlations with calculated disease severity as a function of age and the number of supernumerary CAG repeats, the so-called disease burden score (van Bergen et al., 2016, Zhang et al., 2011) and actual clinical disease severity (Domínguez et al., 2016). These effects were found to be independent from basal ganglia atrophy, suggesting a net increase in total iron in these structures and the potential of iron-sensitive MRI as a biomarker of HD disease progression (Dumas et al., 2012).

Although these studies did show the relevance of iron-sensitive MRI scans in HD, the underlying pathological substrates have never been investigated. Several studies reported the high correlation between MRI and iron levels using quantitative and qualitative techniques in both healthy and diseased brain (Bulk et al., 2018, Fukunaga et al., 2010, Langkammer et al., 2012, Stüber et al., 2014, Sun et al., 2015, Wallace et al., 2016, Hametner et al., 2018). However, the exact pattern of iron accumulation, the colocalization with specific cells and correlation with MRI in HD remains to be elucidated. To date, only one postmortem study linked microglial activation with iron accumulation, suggesting that iron accumulation in HD is predominantly associated with neuroinflammation (Simmons et al., 2007).

Therefore, the objective of this study was to gain more insight into the histopathological correlates of the well-known contrast changes on T2*-weighted MRI in the striatum of HD. We performed ultra-high field *ex vivo* MRI and histopathology on postmortem tissue of the striatum of HD patients to further delineate the pattern of iron accumulation and the colocalization of iron with specific cells known to be associated with HD pathology such as microglia and astrocytes (Crotti and Glass, 2015).

## 2 Methods

### 2.1 Study design

**Fig. 1:**
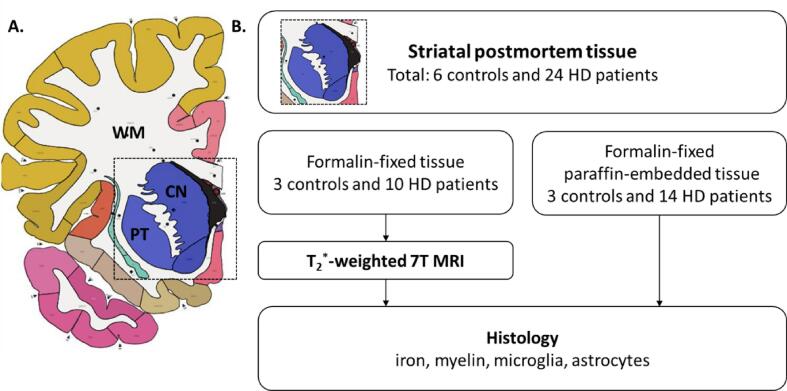
Schematic overview of the study design. From coronal formalin-fixed brain slabs (A), small tissue blocks including the striatum (level of the caudate nucleus and putamen) were dissected (dashed box in A, upper panel in B). Formalin-fixed tissue of three control subjects and 10 HD patients was included for both MRI and histological examination. Formalin-fixed paraffin-embedded tissue of three control subjects and 14 HD patients was selected for histology only. Histological stainings for iron, myelin, microglia and astrocytes were performed, microscopically examined and correlated with the MRI images. Anatomical atlas modified from the Allen Reference Atlas (human atlas, whole brain, 34 years) (Ding et al., 2016). WM = white matter; CN = caudate nucleus; PT = putamen.

Formalin-fixed postmortem brain material of the striatum (including the caudate nucleus and putamen, see Fig. 1) of three control subjects and 10 HD patients was included for both MRI and histological examination. In addition, formalin-fixed paraffin-embedded material of three control subjects and 14 HD patients was selected for only histology to investigate the cellular localization of iron. Histological stainings for iron, myelin, microglia and astrocytes were performed, microscopically examined and correlated with the MRI images.

### 2.2 Postmortem brain material

Formalin-fixed striatal tissue blocks of six control subjects and 24 HD patients was included (Table 1 for characteristics). HD material was selected from the local neuropathology tissue collection of the Leiden University Medical Center (The Netherlands). Brain material from normal controls was selected from the Netherlands Brain Bank (NBB, Netherlands Institute for Neuroscience Amsterdam, The Netherlands) and the Normal Aging Brain Collection (Amsterdam Neuroscience, Amsterdam UMC, Vrije Universiteit Amsterdam, The Netherlands). Brain dissection protocols of the three repositories are identical. Postmortem interval was not significantly different between HD patients and controls (p = 0.182). Anonymity of all subjects was preserved by using a coded system for the tissue samples following the Dutch national ethical guidelines (Code for Proper Secondary Use of Human Tissue, Dutch Federation of Medical Scientific Societies).

**Table 1:** Case characteristics of controls and HD patients included.

	Controls (n = 6)	HD (n = 24)
Age of death, mean ± SD (range)	55.8 ± 5.4 (49–63)	58.5 ± 9.6 (36–74)
Male/Female	2/4	6/18
CAG repeat length, mean ± SD (range)	n.a.	45.4 ± 4.7 (36–57)
Vonsattel grade, (n)	n.a.	2.8 ± 0.8 (1–4)
Postmortem interval in hours, mean ± SD	8.4 ± 1.7	11.2 ± 4.8

### 2.3 Postmortem MRI acquisition

As previous studies showed the potential effect of formalin fixation on absolute iron concentrations and tissue integrity, only material with a maximum fixation duration of four years was selected for postmortem 7T MRI (van Duijn et al., 2011, Schrag et al., 2010). No significant differences in the duration of formalin fixation were found between tissue from HD patients and controls (3.2 years for HD tissue, 1.5 years for control tissue: p = 0.133). The selected tissue blocks were put in a 50 ml tube (Greiner Bio-One). Before MRI the relaxation parameters were partially restored by rinsing the tissue with phosphate buffered saline (PBS) and soaking the tissue in PBS for 24 h (Shepherd et al., 2009). Before scanning, PBS was replaced with an MRI-invisible proton-free fluid (Fomblin LC08, Solvay). Care was taken to avoid the inclusion of air bubbles.

MRI scans were performed on a 7T horizontal bore Bruker MRI system equipped with a 38 mm transmit-receive volume coil and Paravision 6.0.1 imaging software (Bruker Biospin, Ettlingen, Germany). For each tissue block, a 3D multiple gradient echo scan with a total scan time of 10.5 h was acquired. Each brain sample was scanned with the following parameters: bandwidth 10,000 Hz, repetition time 75 ms, echo times 12.5/16.8/21.2/25.5 ms, flip angle 25° at 100 µm isotropic resolution with 14 signal averages. A correction for center frequency drift was applied and only the echoes created with the positive polarity were acquired. The acquisition order was as following, from inner to outer loops: echo loop, slice loop, phase encoding, repetitions. The first echo (TE 12.5 ms) was selected for visual examination because these magnitude images had the best contrast compared with the other echoes.

### 2.4 Histology

The same tissue blocks as used for MRI were embedded in paraffin, stained for iron and myelin and used for macroscopic colocalization between MRI and histology. As markers for microglia did not perform reliably on these sections, possibly due to long fixation durations (>1 year), adjacent tissue blocks from the same area which had been embedded in paraffin shortly after fixation were used for histology and microscopic examination. In addition, the case series for histological examination (without MRI) were expanded with formalin-fixed paraffin-embedded tissue blocks from three control subjects and 14 HD patients. In total, tissue from six controls and 24 HD patients was included for histological examination. The fixation duration from these tissue blocks varied from 10 days till 112 days and no significant differences in fixation duration were found between HD patients and controls (35 days for HD tissue, 38 days for control tissue: p = 0.832). Also, no effect of fixation duration was found on the quality of the stainings after visual inspection of the staining and morphology of cells.

All tissue blocks were serially cut into 5 µm-thick sections. Histochemical detection of iron was done following an adapted version of the Meguro protocol (Meguro et al., 2007, van Duijn et al., 2013). In short, after deparaffinization sections were incubated for 30 min in 1% potassium ferrocyanide, washed followed by 60 min incubation in methanol with 0.01 M NaN_3_ and 0.3% H_2_O_2_. Subsequently, sections were washed with 0.1 M phosphate buffer followed by 30 min incubation in a solution containing 0.025% 3′3-diaminobenzidine-tetrahydrochloride (DAB, DakoCytomation) and 0.005% H_2_O_2_ in 0.1 M phosphate buffer. The reaction was stopped by washing.

Next, for each subject three consecutive sections adjacent to the section stained for iron were used for immunohistochemistry to visualize either myelin, microglia or astrocytes with an anti-proteolipid protein (PLP) monoclonal mouse antibody (1:1000; Serotec), an anti-Iba1 polyclonal rabbit antibody (1:1000; Wako Chemicals USA), or an anti-human GFAP monoclonal mouse antibody (6F2; 1:1000; DakoCytomation, Glostrup, Denmark), respectively. To this end, after deparaffinization all sections were treated with 0.3% H_2_O_2_ in methanol to block endogenous peroxidase activity. Depending on the primary antibody, an antigen retrieval step followed. To detect Iba-1, sections were boiled in EDTA, pH 8.5, for 15 min and cooled down for 1 h. Sections stained for GFAP were boiled in citrate buffer, pH 6, for 15 min and cooled down for 1 h. Primary antibodies were diluted in 1% bovine serum albumin (BSA) in PBS and incubated overnight at room temperature. Secondary antibodies, for PLP and GFAP: biotinylated rabbit anti-mouse (1:200; DakoCytomation, Glostrup, Denmark) and for Iba-1: biotinylated swine anti-rabbit (1:400; DakoCytomation, Glostrup, Denmark), were incubated for one hour at room temperature followed by a 30 min incubation with avidin–biotin complex (ABC, diluted as recommended by the manufacturer, Vector Labs, CA, USA). Signal enhancement was completed by immersion in DAB solution. The sections were counterstained with Harris Haematoxylin, dehydrated, cleared and mounted with Entallan (Merck, Darmstadt, Germany). The slides were digitized using an automatic bright field microscope (Philips Ultra Fast Scanner, Philips, Netherlands) for microscopic evaluation.

Per subject, the caudate nucleus and putamen were scored by two experienced observers (MB and BK) for the presence of enlarged perivascular spaces (absent or present), microglia morphology (homeostatic, dystrophic, mixed dystrophic or deplete) and presence of reactive astrogliosis (absent or present). Observers were blinded to diagnosis and a consensus score per category per subject was reached. Cases were discussed till consensus was reached. Microglia morphology was based on the description by Streit et al. (2014). Homeostatic microglia morphology was defined as microglia with thin, highly branched processes. Dystrophic microglia were observed as a range of dystrophic entities from moderate beaded and twisted processes to complete fragmentation of cells. Mixed dystrophic was defined by a combination of dystrophic microglia and microglia with thicker soma and branches (probably activated microglia). Iba-1 depletion of microglia was scored when only a single cell or very few cells could be observed, and when the majority of the tissue was completely devoid of staining. Classic homeostatic astrocyte morphology was characterized by a dense network of finely branched processes. Marked reactive astrogliosis was observable as a dramatic change in morphology, including prominent hypertrophy and altered ramifications. Representative images of the different morphologies are included in Fig. 5.

### 2.5 MRI – histology comparison

For three control subjects and 10 HD patients the same formalin-fixed tissue block was used for both T2*-weighted 7T MRI and histological examination to colocalize the MRI findings with histology. The most similar MRI slice with respect to the given histology was selected to facilitate their comparison. This was determined based on the physical location of the section in the tissue block, measured by counting the number of sections taken starting at the block surface. At this approximate location, the most similar MRI slice was chosen by visual comparing clearly detectable landmarks (contours, vasculature, tears and so forth).

### 2.6 Statistics

Demographic characteristics and duration of formalin fixation were normally distributed and compared using independent *t*-test and chi-square tests. All statistical analyses were performed with SPSS version 25.0 for Windows (IBM SPSS statistics, Chicago, IL, USA).

## 3 Results

### 3.1 Striatal T2*-weighted MRI contrast is changed in HD

**Fig. 2:**
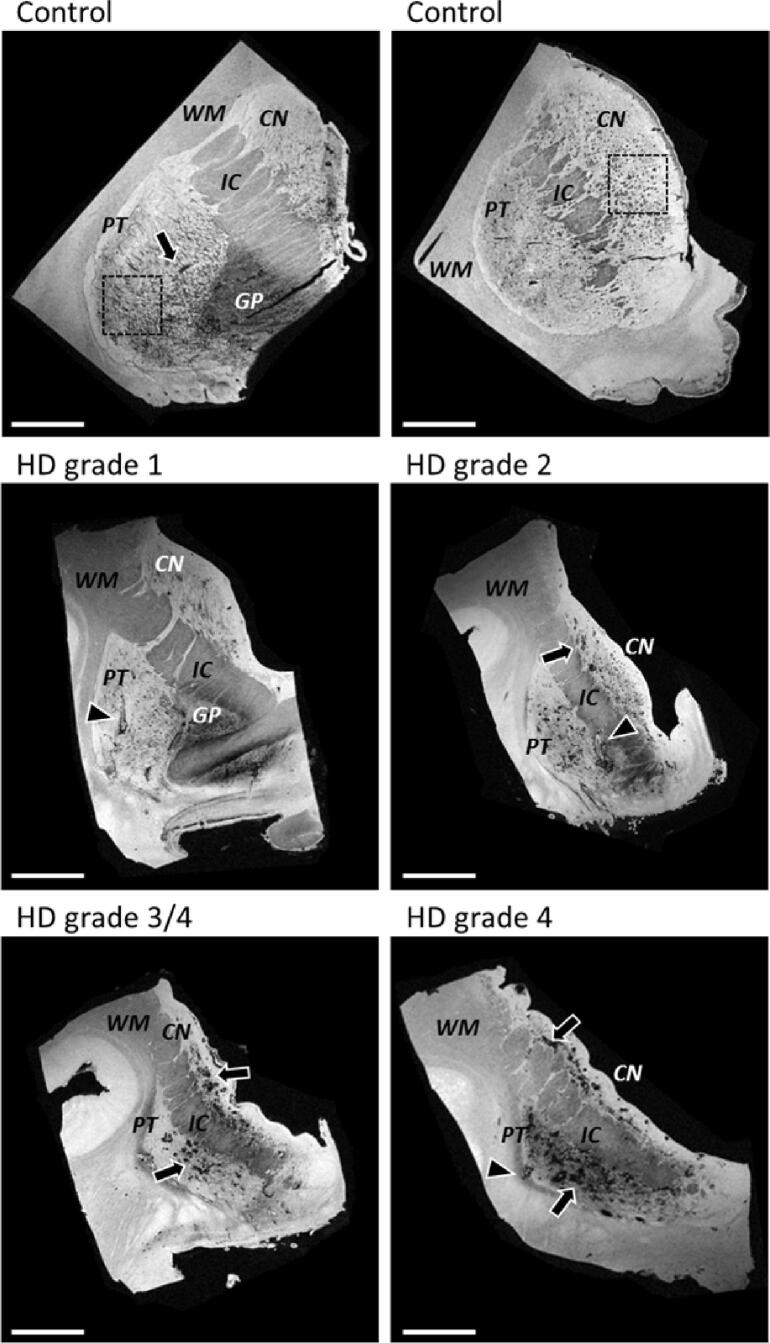
Representative images of T2*-weighted MRI of striatal tissue blocks of two controls and four HD cases. Gross examination of the HD patients showed a varying degree of atrophy which is also represented by the neuropathological severity as indicated by the Vonsattel grade. Small focal hypointensities were found within the caudate nucleus and putamen in control subjects (example within box, but also outside box present). In HD, both the caudate nucleus and putamen showed larger focal hypointensities, which were only rarely observed in control subjects. These larger focal hypointensities sometimes completely lacked any signal (arrow), and were sometimes filled with a hyperintense core probably as a result of residual PBS trapped within these spaces (arrow head). In general, the HD striata were atrophic and more hypointense compared to control striate in line with previously reported *in vivo* findings. CN = caudate nucleus; PT = putamen; IC = internal capsule; GP = globus pallidus; WM = white matter. Scale bar = 1 cm.

Previous *in vivo* studies showed altered T2*-weighted MRI contrast indicative of iron accumulation within the striatum of HD patients compared to controls. We first confirmed these *in vivo* findings by examining the striatal anatomy and T2*-weighted MRI contrast of postmortem tissue from control subject and HD patients. Gross examination of the striatum of all HD patients showed a varying degree of atrophy compared to controls, also represented by the neuropathological severity as indicated by the Vonsattel grade (Fig. 2).

As shown in Fig. 2, on T2*-weighted MRI the anatomy of the striatum was well visible, with clear contrast differences between the substructures of the striatum. The myelin-rich white matter and internal capsule were visible as most hypointense, followed by the caudate nucleus and putamen. In some cases, the globus pallidus was included in the tissue block and could be observed as most hypointense, with even lower signal intensities than the white matter, caudate nucleus and putamen.

Additionally, specifically in control subjects, small focal hypointensities were observed within the caudate nucleus and putamen (Fig. 2, box). HD patients also showed lowest signal amplitude in the myelin-rich areas of the white matter, however, both the caudate nucleus and putamen showed in addition to the small focal hypointensities also larger focal hypointensities, which were only rarely observed in control subjects. These large focal hypointensities sometimes completely lacked signal (Fig. 2, arrow), and sometimes filled with a hyperintense core probably as a result of residual PBS trapped within these spaces (Fig. 2, arrow head). In general, the striatum of HD patients was atrophic and due to the large hypointensities more hypointense compared to control subjects, as previously reported for *in vivo* MRI.

### 3.2 Macroscopic colocalization of iron, myelin, blood vessels and MRI

**Fig. 3:**
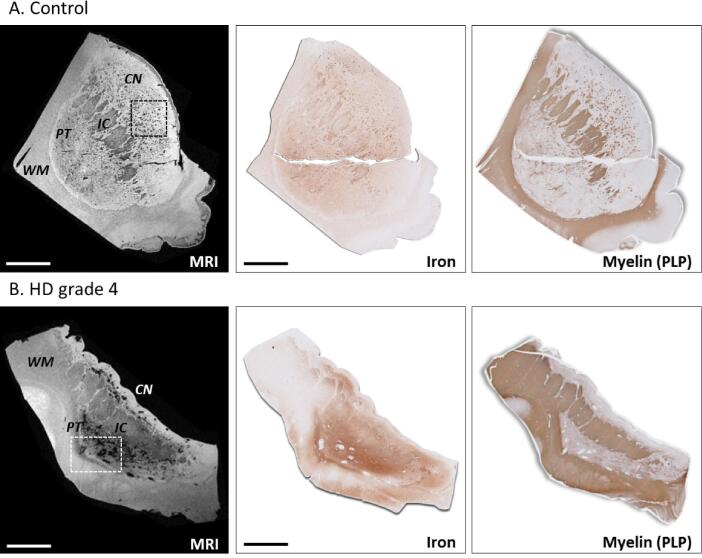
Macroscopic colocalization of MRI and histological staining for iron and immunohistological visualization of myelin. (A) Control subject showed macroscopic colocalization of the small focal hypointensities on MRI with small regions of increased staining intensity in both the iron and myelin staining. (B) HD patients exhibited larger focal hypointensities within the caudate nucleus and putamen. Histology showed high iron, but low myelin staining intensity within these regions. The hypointensities as observed on MRI frequently colocalized with vessels and enlarged perivascular spaces. See Fig. 4 for detailed information of the squares indicated. CN = caudate nucleus; PT = putamen; IC = internal capsule; WM = white matter. Scale bar = 1 cm.

**Fig. 4:**
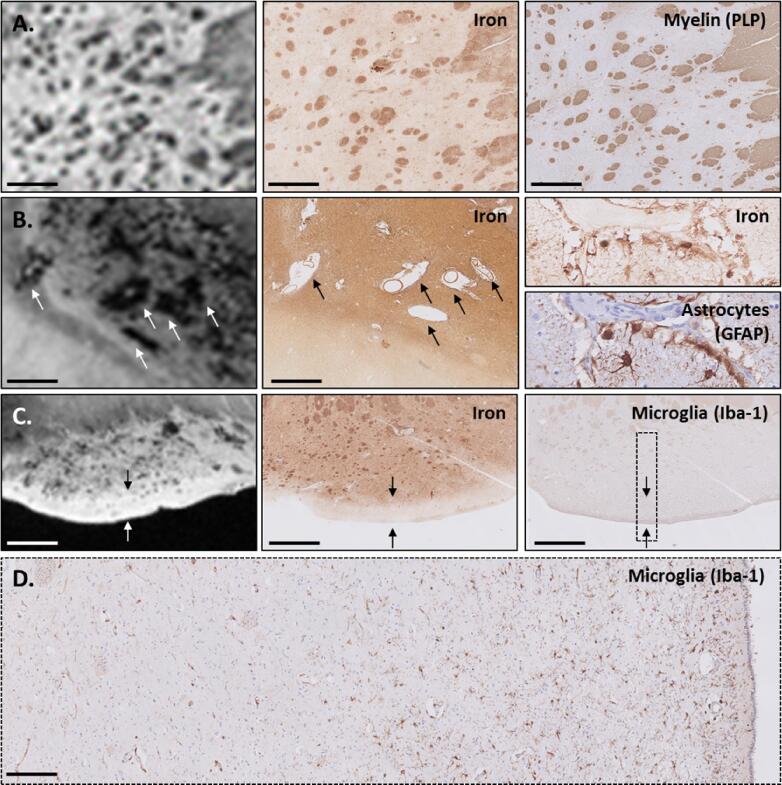
Macroscopic structures affecting the MRI contrast in control subjects and HD patients. (A) Small focal hypointensities on MRI originated from myelinated fiber bundles traversing the caudate nucleus and putamen, which were also characterized by high iron concentrations. (B) Within HD patients, large hypointense regions were found (arrows), frequently colocalized with vessels and enlarged perivascular spaces. The vessel walls were iron rich and surrounded by iron-accumulating astrocytes. (C) The hyperintense border (arrows), known as the subventricular zone, colocalizes with low iron staining intensity and (D) local infiltration of dystrophic and activated microglia. In control subjects only a very thin hyperintense line could be observed. Scale bar in A = 2 mm; Scale bar in B = 1 mm; Scale bar in C = 200 µm.

Histological sections stained for iron and myelin were investigated to establish the origin of the observed MRI contrast changes (Fig. 3, Fig. 4). Macroscopically, the iron staining showed highest staining intensity in the white matter, internal capsule followed by the caudate and the putamen in both control subjects and HD patients. Comparison of MRI with the iron and myelin staining in control subjects showed that the small focal hypointensities on MRI originated from myelinated fiber bundles traversing the caudate nucleus and putamen. These myelinated fibers were also characterized by high iron concentrations (Fig. 3A and 4A).

Within HD patients, the observed focal hypointensities were considerably larger than the small hypointense dots representing fibre bundles. Histological examination revealed that these larger focal hypointensities frequently colocalized with grossly enlarged perivascular spaces of the lenticulostriate arteries. Enlarged perivascular spaces were observed in most HD patients (Table 2, 20/24), but only in two control subjects (2/6) as shown by the absence of large focal hyperintensities on MRI. This was confirmed by histology (Fig. 2, Fig. 3). As can be seen in Fig. 4B, these vessels were characterized by iron-rich vessel walls and iron-accumulating astrocytes surrounding the vessel.

Additionally, we observed that the border of the caudate nucleus, known as the subventricular zone, was hyperintense on MRI and showed low iron staining intensity on histology (Fig. 4C). This was most prominent in HD patients as the subventricular zone was large compared to the severely atrophic caudate nucleus. In control subjects only a very thin hyperintense line could be observed. Microscopic examination showed that this region was characterized by infiltration of dystrophic and activated microglia in both HD patients and control subjects (Fig. 4D).

### 3.3 Colocalization of iron within reactive astrocytes in HD

Macroscopically, the MRI contrast was best explained by the presence of iron, myelin fiber bundles and enlarged perivascular spaces. Stainings for microglia and astrocytes were examined to determine cellular localization of iron.

**Fig. 5:**
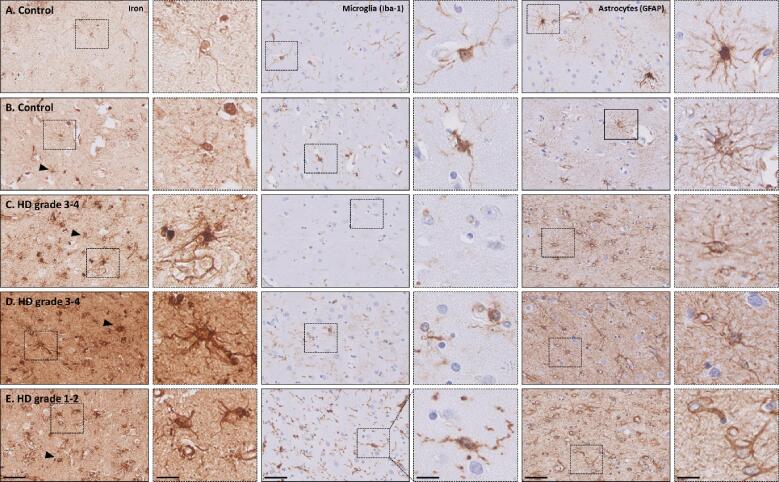
Representative examples of the iron, microglia and astrocyte staining in two control and three HD striata. Microglia staining showed a variety of morphologies within both control subjects and HD patients, ranging from (A, putamen) homeostatic microglial morphology to (B, putamen & D, putamen) dystrophic morphology or (E, caudate nucleus) mixed dystrophic, defined as a mixture of both dystrophic and activated microglia. (C, caudate nucleus) Microglia depletion was also frequently observed. (C-E) In all HD patients, marked reactive astrogliosis was observable as dramatic change in morphology including hypertrophic and altered ramifications. Interestingly, the iron staining showed a very similar pattern as the staining for astrocytes. No clear differences in iron staining were found between HD patients with deplete, dystrophic or a mix of activated and dystrophic microglia as the iron staining seemed to be dominated by iron accumulation within astrocytes. No correlation between iron accumulation as well as morphological changes in microglia and astrocytes with the Vonsattel grade was found. Scale bar = 50 µm; Scale bar in zoom (indicated with dotted lines) = 25 µm.

In most control subjects (4/6), predominantly homeostatic microglia were observed in both the caudate nucleus and putamen (Fig. 5A & Table 2). Two cases showed microglia with a range of dystrophic entities from moderate beaded and twisted processes to complete fragmentation of cells (Fig. 5B). Astrocytes showed in all control subjects a classic homeostatic morphology characterized by a dense network of finely branching processes and were especially found close to vessels. Within the iron staining, we could occasionally identify cells closely resembling microglia and astrocytes. No differences regarding the iron staining could be observed between cases with homeostatic or dystrophic microglia (Fig. 5A&B).

**Table 2:** Overview of microscopic results of enlarged perivascular spaces, microglia and astrocyte morphological examination per subject. C = Caudate nucleus; P = Putamen; C-P = caudate nucleus and putamen.

	Age of death	Vonsattel	CAG repeat length	Cause of death	Enlarged perivascular spaces	Microglia morphology				Astrogliosis
						Homeostatic	Deplete	Dystrophic	Mixed dystrophic	
HD#1	74	1	36	Euthanasia	C-P			P	C	C-P
HD#2	59	1–2	43	Pneumonia, end stage HD			C-P			C-P
HD#3	64	1–2	44	Unknown	C-P		C-P			C-P
HD#4	46	2	52	Pneumonia	C-P		C	P		C-P
HD#5	60	2	42	Euthanasia	C-P		C-P			C-P
HD#6	47	2–3	46	Pneumonia				C-P		C-P
HD#7	71	2–3	43	Cerebrovasculair accident, pneumonia	C-P				C-P	C-P
HD#8	60	2–3	45	Cachexia, end stage HD					C-P	C-P
HD#9	65	2–3	43	End stage HD	C-P		C		P	C-P
HD#10	61	3	47	End stage HD	C-P		P	C		C-P
HD#11	72	3	42	Septic after urinary tract infection	P	P		C		C-P
HD#12	60	3	46	Dehydration, end stage HD	C-P		C-P			C-P
HD#13	65	3	44	End stage HD	C-P		C-P			C-P
HD#14	51	3	46	Dehydration, cachexia	C-P				C-P	C-P
HD#15	55	3	not available	End stage HD	C-P		C-P			C-P
HD#16	56	3	42	Euthanasia	P			C-P		C-P
HD#17	36	3	51	Pneumonia	C-P			C-P		C-P
HD#18	68	3–4	43	Pneumonia, end stage HD				C-P		C-P
HD#19	71	3–4	43	Cachexia, end stage HD	C-P				C-P	C-P
HD#20	57	3–4	44	Cachexia, respiratory infection	C-P				C-P	C-P
HD#21	44	3–4	56	End stage HD	C-P		C-P			C-P
HD#22	60	4	not available	Dehydration, cachexia	C-P		C-P			C-P
HD#23	46	4	57	End stage HD	C-P		C-P			C-P
HD#24	57	4	43	Pneumonia	C-P		C-P			C-P
Control#1	63	–	–	Euthanasia	P	C-P				Absent
Control#2	59	–	–	Euthanasia	Absent	C-P				Absent
Control#3	51	–	–	Suicide by withering	Absent	C-P				Absent
Control#4	61	–	–	Pneumonia, cachexia	Absent			P		Absent
Control#5	49	–	–	Sudden death	P		C	P		Absent
Control#6	52	–	–	Euthanasia	Absent	C		P		Absent

Within the HD cohort, microglia showed a larger morphological variety compared to control subjects (Table 2). Whereas in all control subjects microglia could be detected, 50% (13/24) of the HD patients showed very few microglia, an observation defined as microglia depletion (10/24) (Fig. 5C). In these cases, only a single cell or a very few cells could be observed within the entire tissue section and the majority of the tissue within both the caudate nucleus and putamen was completely devoid of Iba-1 staining. Some HD patients showed a combination of microglia depletion and presence of dystrophic microglia (3/24). Other cases were characterized predominantly by dystrophic microglia (4/24) (Fig. 5D), or by a mixture of both dystrophic microglia and microglia with thicker branches (probably activated microglia), defined as mixed dystrophic morphology (6/24) (Fig. 5E). Marked reactive astrogliosis was present in all HD patients, observable as a dramatic change in morphology, including prominent hypertrophy and altered ramifications (Fig. 5C-E).

The iron staining showed a very similar pattern to the staining for astrocytes. Apart from a general increase in the background intensity, iron was predominantly found in cells morphologically resembling reactive astrocytes. Although the morphological changes of microglia varied across patients, no clear differences in iron staining were found between patients with deplete, dystrophic or a mix of activated and dystrophic microglia, as the iron staining seemed to be dominated by iron accumulation within astrocytes.

The morphological changes in microglia and astrocytes were not correlated with the neuropathological severity as indicated by the Vonsattel grades. Depleted, dystrophic, and activated microglia were found in HD patients with low as well as with high Vonsattel grades. In addition, no association was found with CAG repeat length, age of death or cause of death.

### 3.4 Histopathological correlates of areas without MRI contrast changes

**Fig. 6:**
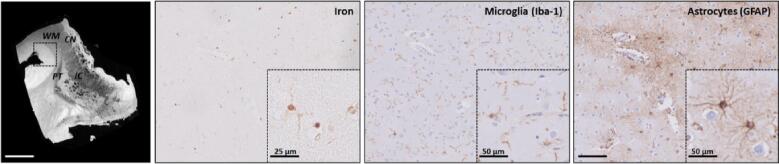
Region without MRI contrast changes showing normal astrocyte morphology and less intense iron staining. Within HD, the neocortex showed high signal intensity on MRI and low iron staining intensity on histology. Microscopic examination of this region showed in this specific case the presence of dystrophic microglia. However, astrocytes showed a normal morphology which possibly reflects that the region is less affected by the disease. Within the iron staining significantly less background was observed and occasionally we could identify iron-positive cells morphologically resembling microglia and astrocytes. CN = caudate nucleus; PT = putamen; IC = internal capsule; WM = white matter. Scale bar in MRI = 1 cm; Scale bar in histology = 100 µm; See zoom for scale bar size.

To confirm iron accumulation within reactive astrocytes as the predominant source of the general increase of iron within the striatum and hence the observed postmortem MRI contrast changes, other regions showing no abnormalities on MRI were further investigated (Fig. 6).

In the HD brain, the neocortex showed high signal intensity upon MRI and low iron staining intensity on histology. Microscopic examination of this region showed the presence of dystrophic microglia. However, in contrast to the caudate nucleus and putamen, astrocytes were characterized by a fine network of branches and thus showed significantly less dramatic changes suggesting that this cortical region is less affected by the disease. Within the iron staining, significantly less background was observed and occasionally we could identify iron-positive cells morphologically resembling microglia and astrocytes.

## 4 Discussion

This study aimed to gain insight into the histopathological correlates of the previously reported T2*-weighted MRI contrast changes in the striatum in HD. Ultra-high field *ex vivo* MRI showed that the striatum of HD patients was characterized by large focal hypointense regions that frequently colocalized with enlarged perivascular spaces. Histopathology showed that reactive astrocytes are the predominant source of the general increase of iron within the striatum and hence the observed postmortem MRI contrast changes.

As already hypothesized by previous *in vivo* MRI studies showing T2*-weighted MRI changes within the striatum of HD patients (Domínguez D et al., 2016, Jurgens et al., 2010, van Bergen et al., 2016, Vymazal et al., 2007, Langkammer et al., 2012), the spatial colocalizations reported in this study indeed demonstrate a relationship between T2*-weighted MRI and increased iron accumulation. Apart from an increase of diffuse iron within the parenchyma, we showed that on *ex vivo* MRI a significant part of the MRI contrast changes is caused by enlarged perivascular spaces causing large focal hypointensities. Vessels within the striatum were positive for iron, which corresponds to previous reports on both healthy ageing as well as on neurodegenerative diseases (Casanova and Araque, 2003, Morris et al., 1992). It is suggested that the high metabolic rate of the basal ganglia makes this structure more prone to the accumulation of minerals as calcium and iron (Casanova and Araque, 2003). Although being clearly a factor for MRI contrast changes *ex vivo*, evidence for the presence of enlarged perivascular spaces from *in vivo* HD studies is currently, apart from one study (Valdés Hernández et al., 2019), lacking. In contrast, several studies reported increased brain vessel density in both mice and patients with HD with specifically an increased proportion of small compared to medium‐sized blood vessels within the putamen (Drouin-Ouellet et al., 2015, Franciosi et al., 2012, Lin et al., 2013). At this moment, we cannot exclude the possibility that the observed enlarged perivascular spaces are a postmortem artefact, e.g., pronounced parenchymal shrinkage of these structures upon fixation, due to a loss of tissue integrity as a result of neurodegeneration. Therefore, *in vivo* validation with T2-weighted methods are needed to further investigate this phenomenon.

Although the presence of enlarged perivascular spaces does need *in vivo* validation, the additional observation of increased iron within astrocytes, iron-rich vessel walls and iron-accumulating astrocytes surrounding the vessels *ex vivo* does, at least partially, explain the increased iron signal in these regions already demonstrated upon *in vivo* MRI. Should the enlarged perivascular spaces observed *ex vivo* actually represent fixation artefacts, the iron accumulation within vessel walls and astrocytes surrounding the vessel will result in faster MRI signal decay and thus a darker appearance of the striatum on *in vivo* MRI in HD patients compared to controls. In contrast, existence of the enlarged perivascular spaces *in vivo* with interstitial fluid trapped within the these compartments will give a high MRI signal on *in vivo* T2 or T2*-weighted scans, which in combination with the low signal due to the surrounding iron-rich vessel walls and iron-accumulating astrocytes will results in a “salt and pepper”-like appearance of these areas with adjacent hypo- and hyperintense signals. However, we expect that the net increase and decrease of MRI signal in the vicinity of the enlarged perivascular spaces is not detectable on *in vivo* MRI due to partial volume effects as a consequence of scanning at much lower resolution compared to the *ex vivo* findings. Nevertheless, increased iron within the parenchyma and activated astrocytes will be present resulting in a general darker appearance of the striatum.

Next to these macroscopic structures affecting the MRI contrast, we investigated the cellular localization of iron within microglia and astrocytes, as both are known to be involved in HD pathogenesis (Crotti and Glass, 2015). Positron emission tomography (PET) suggests that microglial activation is present in HD patients and correlates with disease severity and striatal neuronal loss (Pavese et al., 2006). Several studies also showed that microglial activation is an early event in HD pathogenesis as microglial activation is already observed in gene-carriers prior onset of symptoms (Politis et al., 2015, Tai et al., 2007). In contrast, reactive astrocytes have been observed only after neurodegeneration has become evident (Vonsattel et al., 2011, Vonsattel et al., 1985). Importantly, the increased number of reactive astrocytes is spatially correlated with the gradient of neurodegeneration within the striatum (Vonsattel et al., 1985).

We report here that 50% of the HD patients showed microglia depletion rather than dystrophy or activation, and we found no correlation of microglial abnormalities with Vonsattel grades. As microglia within the subventricular zone, internal capsule and cortex were observed within the same brain section of these cases, we do not attribute this observation to a staining artefact. In addition, these findings were confirmed using the antibody TMEM119, a microglial marker that discriminates resident microglia from blood‐derived macrophages (not shown) (Satoh et al., 2016). However, as reported recently by Lier et al. (2019), a subgroup of microglia exist that exhibit a localized loss of Iba-1 in obese subjects, which was in part linked with obesity and hepatic dysfunction. These Iba-1 negative microglia were also negative for ferritin, but remained immuno-positive for markers expressed primarily by microglial cells (GPX1) (Power and Blumbergs, 2009), and intraparenchymal microglia (P2ry12) (Mildner et al., 2017). Whether the Iba-1-negative microglia in our study belong to the same subgroup as described by the Lier needs further investigation. Nevertheless, Lier et al. (2019) also reported that the Iba-1 negative microglia also stained negative for ferritin, corresponding to the observations in the current study that the microglia did not significantly contribute to the iron staining as this was dominated by activated astrocytes. Alternatively, based on the descriptions by Streit et al. (2014), we hypothesize that microglia depletion might be a consequence of prolonged microglia activation, with activated microglia becoming dystrophic and undergoing cytorrhexis. Eventually, only scattered fragments and cell nuclei are left which can be observed as microglia depletion in our study.

We hypothesize that initially in HD, microglia likely play an important role in sequestering iron, but due to prolonged activation, microglia become dystrophic and degenerate. Following microglia activation and neurodegeneration, astrocytes are activated (Liddelow et al., 2017). Upon activation their ability to sequester iron increases. Finally, after microglial degeneration in end stage HD, activated astrocytes take over the role of microglia as the predominant iron-sequestering glial cells. This hypothesis is based on previous studies reporting that microglia are the first responders to iron accumulation within the brain parenchyma and are known to rapidly and effectively sequester excess of iron (Streit et al., 2014) resulting in an iron-positive dystrophic microglial phenotype. This phenomenon has been observed in several neurodegenerative diseases, including HD (Simmons et al., 2007, Jellinger et al., 1990). Astrocytes are known to play a crucial role in iron handling as well by controlling iron uptake through the BBB and redistribution of iron to neuronal cells (Moos et al., 2007). The competence of astrocytes to sequester iron is further increased upon activation in a neuroinflammatory and neurodegenerative environment (Pelizzoni et al., 2013, Rathore et al., 2012). Reversely, studies focusing on the role of systemic iron on monocyte and macrophage activation showed that these cells do not only sequester iron upon activation, but also that cellular labile iron mediates inflammation. More specifically, cellular labile iron can activate the NLRP3 inflammasome, resulting in the secretion of the pro-inflammatory cytokine interleukin 1β and eventually a pro-inflammatory environment (Nakamura et al., 2016). As interleukin 1β also plays an important role in microglia and astrocyte activation (Heneka et al., 2015), increased iron levels could be a cause as well as a consequence of neuroinflammation. A limitation of our study is that the HD material included patients with relatively high Vonsattel grades. Whereas previous studies did report a correlation between microglia activation, reactive astrocytes and disease severity (Pavese et al., 2006, Vonsattel et al., 1985), we were unable to reproduce this link. The availability of tissue from patients with very low disease severity is limited. Consequently, it also remains unclear whether the iron distribution in microglia and astrocytes is different in earlier stages of the disease. Translating our findings to pre-manifest HD patients remains challenging as these patients are expected to have microglia activation, iron accumulation but fewer or no reactive astrocytes. Correlating *in vivo* T2*-weighted MRI with biofluid markers for microglia and astrocytes might help interpret the MRI findings.

In conclusion, we show that compared to control tissues the HD striatum has a distinctive phenotype on T2*-weighted MRI. On *ex vivo* MRI, the contrast changes are heavily biased by enlarged perivascular spaces, from which it is currently unknown whether these are fixation artefacts or a disease-specific observation. Microscopically, iron was predominantly found within reactive astrocytes. Clinically, these results are important for the interpretation and understanding of potential underlying mechanisms of T2*-weighted MRI results in HD patients. However, the exact sequence of iron accumulation within astrocytes and microglia during disease progression, and the implication of glial iron accumulation for disease progression needs to be further investigated.

## Funding

This work was supported by a grant from 10.13039/501100001826ZONMW program Innovative Medical Devices Initiative, project Imaging Dementia: Brain Matters (104003005).

## CRediT authorship contribution statement

**Marjolein Bulk:** Conceptualization, Formal analysis, Investigation, Writing - original draft. **Ingrid Hegeman-Kleinn:** Investigation. **Boyd Kenkhuis:** Investigation. **Ernst Suidgeest:** Investigation. **Willeke Roon-Mom:** Conceptualization. **Jan Lewerenz:** Conceptualization. **Sjoerd Duinen:** Investigation, Conceptualization. **Itamar Ronen:** Conceptualization, Writing - original draft, Supervision, Funding acquisition. **Louise Weerd:** Conceptualization, Writing - original draft, Supervision.

## Declaration of Competing Interest

The authors declare that they have no known competing financial interests or personal relationships that could have appeared to influence the work reported in this paper.
